# Means of Ascertaining If Alcohol Be Perfectly Pure

**Published:** 1847-10

**Authors:** 


9. *Means of ascertaining if Alcohol be perfectly pure.*—M. Cassoria employs the anhydrous sulphate of copper to determine if alcohol contains any water. The salt will remain white, if put in anhydrous alcohol in a well stopped bottle, but will become blue if the alcohol contains any water.—Translated from Journ. de Pharm.—Bulletin Gén. de Therap, for *Ibid*

